# Pompe Disease: A Review of Diagnosis, Molecular Genetics, and Treatment Management

**DOI:** 10.2174/011573403X377990250818051032

**Published:** 2025-09-10

**Authors:** Najlae Adadi, Mohamed Yassine El Brouzi

**Affiliations:** 1 Higher Institute of Nursing Professions and Health Techniques, Dakhla, Morocco;; 2 Laboratory of Biology and Health, Neurosciences, Neuroimmunology and Behaviour Unit, Faculty of Science, Ibn Tofail University, Kenitra, Morocco

**Keywords:** Pompe disease, GAA gene, enzymatic replacement therapy, gene therapy, genetic counseling, vector immunogenicity

## Abstract

**Introduction:**

Pompe disease, a rare autosomal recessive lysosomal storage disorder, results from mutations in the GAA gene that lead to deficient acid alpha-glucosidase activity and glycogen accumulation in various tissues.

**Methods:**

This review employs the diagnostic approach to the disease, encompassing enzymatic assays and molecular genetics, with a focus on genotype-phenotype correlations and region-specific mutations.

**Results:**

Over 500 mutations in the GAA gene, including missense, nonsense, insertions, deletions, and splicing defects, contribute to varying levels of enzyme deficiency, accounting for the diverse clinical manifestations of Pompe disease.

**Discussion:**

Current therapies, including Enzyme replacement therapy (ERT), are the cornerstone treatments for Pompe disease, utilizing recombinant human alpha-glucosidase to restore enzyme activity and reduce glycogen accumulation in lysosomes. While ERT significantly improves survival, cardiomyopathy, and respiratory function, its limited uptake in skeletal muscle and immunogenicity pose challenges. Innovations include immune tolerance protocols and next-generation enzymes to enhance skeletal muscle delivery. Gene therapy emerges as a promising alternative, leveraging viral vectors to deliver functional GAA genes, thereby enabling sustained endogenous enzyme production and addressing limitations of ERT. Preclinical and early-phase trials demonstrate efficacy, reduced immunogenicity, and enhanced skeletal muscle uptake; however, challenges, such as vector immunogenicity and cost, remain. Thus, genetic counseling is essential for family planning and managing emotional and psychosocial challenges related to this disease.

**Conclusion:**

This article highlights advances and challenges in the diagnosis, management, and treatment of Pompe disease, providing a comprehensive resource for clinicians and researchers.

## 1 INTRODUCTION

Pompe disease, also known as glycogen storage disease type II, is an autosomal recessive lysosomal storage disorder caused by a deficiency of the enzyme alpha-glucosidase acid (GAA) [1-3]. This enzyme is essential for the breakdown of glycogen in lysosomes. When GAA activity is reduced or absent, glycogen accumulates abnormally in the lysosomes, leading to cellular dysfunction and progressive myopathy [4]. The clinical presentation of Pompe disease varies significantly, ranging from a severe infantile form to a late-onset form primarily affecting skeletal and respiratory muscles [2]. Its estimated prevalence lies between 1 in 9,000 and 1 in 40,000 individuals [5]. Research into the disease has revealed a genetic basis that underscores its clinical variability, with over 500 mutations identified in the GAA gene.

The infantile-onset form manifests within the first year of life with hypertrophic cardiomyopathy, profound muscular weakness, and respiratory insufficiency. Without treatment, this form is typically fatal by the age of two [6]. Conversely, the late-onset form (LOPD) has a milder phenotype, with symptoms, such as progressive muscle weakness and respiratory compromise, appearing later in life [7]. These patients typically do not exhibit cardiomyopathy, which distinguishes the disease from the infantile form.

Advances in molecular genetics and diagnostic tools have significantly enhanced our ability to confirm a diagnosis of Pompe disease and elucidate its pathophysiology [8, 9]. The identification of mutations in the GAA gene has provided valuable insights into genotype-phenotype correlations, facilitating the development of targeted therapies. One notable case, reported by Adadi *et al*. (2018), highlights the diagnostic challenges in regions with high consanguinity rates. In a Moroccan family with a history of hypertrophic cardiomyopathy and early sudden cardiac deaths, whole-exome sequencing revealed a homozygous deleterious mutation in the GAA gene (c.236_246delCCACACAGTGC, p.Pro79Argfs X13) [5]. The post-mortem diagnosis provided essential information for genetic counseling and prevention in future pregnancies.

The therapeutic landscape for Pompe disease has evolved dramatically over the past two decades [10, 11]. Enzyme replacement therapy (ERT) remains the cornerstone of treatment, particularly for infantile-onset Pompe disease [1, 12]. However, challenges, such as immunogenicity and incomplete efficacy, underscore the need for innovative approaches, including gene therapy. This review aims to provide a comprehensive overview of the diagnosis, molecular genetics, and current therapeutic strategies for Pompe disease, with a particular focus on advances in enzymatic and gene-based therapies.

In this study, we conducted a mini-review focusing on Pompe disease, its diagnosis, molecular genetics, and treatment management. We performed a structured literature search using the databases Scopus, PubMed, Medline, and Google Scholar. Relevant articles published in English were selected based on their relevance to the diagnosis, molecular genetics, and treatment of Pompe disease. We used specific keywords, such as Pompe disease, glycogen storage disease type II, diagnosis, GAA gene, and enzyme replacement therapy. Titles and abstracts were screened to include the most recent and relevant publications, while articles not directly related to the scope of this review were excluded.

## 2 DIAGNOSIS

### 2.1 Clinical Phenotype

Pompe disease encompasses a spectrum of clinical manifestations influenced by the residual activity of the enzyme GAA. The disorder is divided into two main forms: infantile-onset Pompe disease (IOPD) and late-onset Pompe disease (LOPD), each presenting distinct clinical profiles [6, 7, 13].

#### 2.1.1 Infantile-Onset Pompe Disease (IOPD)

IOPD is the most severe form, typically manifesting within the first few months of life [14]. Hypertrophic cardiomyopathy is a hallmark feature, often leading to progressive heart failure. Affected infants exhibit profound hypotonia, which significantly impairs motor development, and respiratory dysfunction due to weakened diaphragm and intercostal muscles. Feeding difficulties and failure to thrive are also common in this form, contributing to poor outcomes. Without treatment, most patients with IOPD succumb to cardiorespiratory failure before the age of two [5, 15, 16].

#### 2.1.2 Late-Onset Pompe Disease (LOPD)

LOPD typically presents with a milder phenotype, often occurring later in childhood, adolescence, or adulthood [17, 18]. Patients experience progressive proximal muscle weakness, particularly in the limb-girdle and axial muscles, which may lead to mobility challenges, such as difficulty climbing stairs or rising from a seated position. Respiratory insufficiency is a key feature, with diaphragmatic weakness contributing to nocturnal hypoventilation and chronic respiratory symptoms. Unlike IOPD, cardiomyopathy is typically absent in LOPD, a critical distinguishing factor. The slow progression of LOPD often results in diagnostic delays, as symptoms may mimic other conditions like inflammatory myopathies or muscular dystrophies [13, 17].

#### 2.1.3 Atypical and Intermediate Presentations

Intermediate forms exhibit overlapping features of IOPD and LOPD. Additionally, novel phenotypes, such as gastrointestinal symptoms and vascular or central nervous system involvement, have been reported in long-term survivors. These atypical manifestations highlight the expanding clinical spectrum of Pompe disease [14, 18-20].

#### 2.1.4 Case Study Insight

A post-mortem diagnosis in a Moroccan family highlighted the severe IOPD phenotype. A seven-month-old girl presented with hypertrophic cardiomyopathy and died suddenly. Whole-exome sequencing revealed a homozygous mutation in the GAA gene, emphasizing the importance of combining clinical, enzymatic, and genetic analyses in diagnosing Pompe disease [5]. Recognizing the diverse clinical phenotypes of Pompe disease is crucial for early diagnosis and initiation of treatment, which can significantly alter the disease course and prognosis.

### 2.2 Medical Analyses

Medical analyses are essential in confirming the diagnosis of Pompe disease, distinguishing between its phenotypes, and assessing the severity of organ involvement. These analyses involve enzymatic assays, imaging studies, and laboratory investigations, supported by advanced genetic testing.

Comprehensive medical analyses are vital for accurately diagnosing Pompe disease and guiding treatment decisions (Table **1**). Enzymatic assays and genetic testing remain the gold standards, complemented by imaging and biochemical studies to evaluate systemic involvement [23].

## 3 MOLECULAR GENETICS

### 3.1 GAA Gene

The GAA gene, located on chromosome **17q25.2-q25.3**, encodes the lysosomal enzyme GAA, which is responsible for the breakdown of glycogen into glucose within lysosomes [19, 24]. Mutations in this gene lead to deficient or absent GAA activity, resulting in the pathological accumulation of glycogen in multiple tissues, including skeletal, smooth, and cardiac muscles. This accumulation underlies the clinical manifestations of Pompe disease [24].

The GAA gene spans approximately 28 kb and consists of 20 exons. It encodes a precursor enzyme of 110 kDa, which undergoes post-translational processing to yield mature active forms of 76 kDa and 70 kDa [24, 25]. These forms are critical for the degradation of lysosomal glycogen. The dysfunction or absence of this enzyme leads to lysosomal swelling, tissue damage, and the progressive symptoms observed in Pompe disease [19, 21].

Over 500 mutations have been identified in the GAA gene, including missense mutations, nonsense mutations, insertions, deletions, and splicing defects [19, 24]. These mutations can cause varying degrees of enzyme deficiency, explaining the broad clinical spectrum of Pompe disease. Common mutations include the following:

- **c.-32-13T>G (IVS1):** It is a splicing mutation frequently associated with late-onset Pompe disease [7, 26].
- **c.525delT:** It is a frameshift mutation, leading to severe phenotypes in infantile-onset Pompe disease [27].
- **c.236_246delCCACACAGTGC:** It is a deletion mutation identified in a Moroccan family, linked to severe infantile-onset disease [5, 22].
- Certain mutations correlate with specific clinical phenotypes. For example:
- Severe loss-of-function mutations, such as nonsense or frameshift mutations, are typically associated with the infantile-onset form of the disease.
- Mutations that allow partial residual enzyme activity, such as splicing mutations, are more common in late-onset forms [13, 17].

Some mutations in the GAA gene exhibit ethnic or geographical specificity. For example, the mutation **c.236_246delCCACACAGTGC**, reported in a Moroccan family, highlights the importance of understanding regional genetic variations for effective diagnosis and genetic counseling [5]. Identifying specific mutations in the GAA gene is essential for:

1. **Confirming diagnosis:** Genetic testing complements enzymatic assays, especially in atypical or ambiguous cases [19, 24].
2. **Genetic counseling:** Mutation analysis provides information about inheritance patterns and recurrence risks [24].
3. **Therapeutic development:** Understanding the molecular defects enables the design of targeted therapies, such as gene therapy and personalized approaches [16, 22].

### 3.2 Enzymatic Assay

The enzymatic assay for GAA activity is fundamental in diagnosing Pompe disease. It provides a direct measurement of the enzyme's function, confirming the biochemical basis of the disorder. The assay utilizes biological samples, such as dried blood spots, leukocytes, fibroblasts, or muscle biopsies, to evaluate GAA activity. Synthetic substrates like 4-methylumbelliferyl-alpha-D-glucopyranoside are used in this process, and the release of a fluorescent product correlates with enzyme activity [13, 21].

Dried blood spots are a common initial screening method due to their convenience. Confirmatory tests are performed using leukocytes or fibroblasts, which provide higher specificity. In rare instances, muscle biopsies are utilized, although advancements in non-invasive techniques have largely replaced this approach [16, 19]. The interpretation of GAA activity levels differentiates between disease types. In IOPD, GAA activity is nearly absent, whereas LOPD exhibits residual activity, typically between 2-30% of normal. Carriers show intermediate activity levels without clinical manifestations.

Challenges in enzymatic assays include pseudodeficiency alleles, particularly in Asian populations, which reduce GAA activity without causing disease. These require genetic testing to distinguish. Occasionally, reduced GAA activity may overlap with findings in other lysosomal disorders, necessitating further testing for accurate diagnosis [17, 22].

Enzymatic assays are frequently complemented by genetic testing to identify pathogenic variants and corroborate findings. Elevated serum creatine kinase, alanine aminotransferase, and aspartate aminotransferase levels serve as additional biochemical markers, supporting enzymatic results. Imaging studies and electrophysiological assessments further aid in evaluating systemic involvement.

As mentioned earlier, in a Moroccan family, an enzymatic assay revealed severely reduced GAA activity in an infant with hypertrophic cardiomyopathy. This finding, combined with genetic testing, confirmed the diagnosis of IOPD and emphasized the assay's diagnostic value in severe phenotypes [5]. Overall, the enzymatic assay remains an indispensable diagnostic tool, ensuring rapid and reliable detection of Pompe disease when integrated with genetic and clinical analyses.

**Fig. (1):**
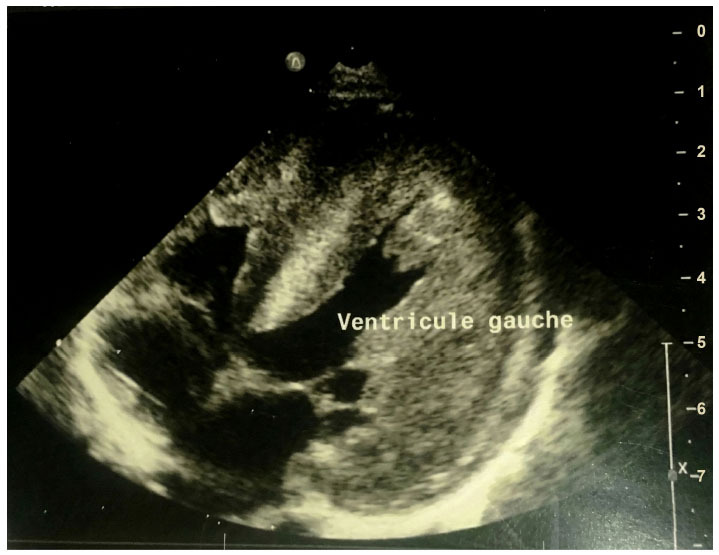
Echocardiography of the LV, parasternal short-axis views. The severe biventricular hypertrophy at diagnosis.

Speckle tracking echocardiography (STE) represents a valuable advancement in the cardiac assessment of patients with Pompe disease, particularly those with infantile-onset forms characterized by early and progressive myocardial involvement (Fig. **1**) (Adadi *et al*., 2018). Unlike conventional echocardiography, STE enables the quantification of myocardial deformation (strain) across all cardiac chambers, allowing clinicians to detect subtle changes in myocardial function even before the appearance of overt structural abnormalities, such as left ventricular hypertrophy. This early identification of subclinical myocardial dysfunction is critical in Pompe disease, where timely therapeutic intervention can significantly impact clinical outcomes. By providing incremental diagnostic and prognostic information, STE has the potential to enhance routine cardiac monitoring, guide treatment decisions, and facilitate more precise risk stratification in both pediatric and adult populations affected by this lysosomal storage disorder [5].

### 3.3 Mutations of the GAA Gene

The GAA gene is highly polymorphic, with more than 500 mutations identified to date [25, 28, 29]. These mutations result in varying degrees of enzyme deficiency, which correlate with the clinical severity of Pompe disease. Mutations in the GAA gene include missense mutations, nonsense mutations, splicing defects, insertions, deletions, and complex rearrangements [2, 24]. The nature of the mutation often determines whether the disease manifests as the severe infantile-onset form (IOPD) or the milder late-onset form (LOPD) [7, 25].

Missense mutations lead to single amino acid changes, potentially disrupting the structure and function of the GAA protein. For instance, the **p.Asp645Glu** mutation has been associated with residual enzyme activity and a milder phenotype. In contrast, nonsense mutations such as **p.Arg854X** result in truncated, nonfunctional enzymes, typically causing severe disease [19, 22, 30].

Splicing mutations, such as **c.-32-13T>G** (IVS1 mutation), are among the most common in Pompe disease and are strongly associated with LOPD. These mutations reduce but do not eliminate enzyme activity, contributing to later-onset symptoms [17, 31].

Frameshift mutations, such as **c.525delT**, and large deletions often lead to complete loss of enzyme activity [27]. For example, the Moroccan family mentioned earlier, with IOPD, was found to have a homozygous deletion, **c.236_246delCCACACAGTGC**, which disrupted the reading frame and resulted in severe disease [5]. Such mutations are frequently associated with the infantile-onset phenotype due to their profound impact on enzyme function.

Mutations that allow some residual GAA activity, such as splicing defects, are commonly associated with LOPD. Conversely, null mutations (*e.g.,* nonsense mutations or large deletions) are associated with IOPD. This correlation is critical for predicting disease severity, guiding clinical management, and determining therapeutic interventions [13, 16].

Certain mutations show a geographical or ethnic distribution. For example, the **c.-32-13T>G** mutation is prevalent in Caucasian populations, while specific deletions, such as **c.236_246delCCACACAGTGC**, have been reported in Moroccan families. Such findings highlight the importance of population-specific genetic studies for accurate diagnosis and effective genetic counseling, as reported earlier [5].

Identifying specific mutations in the GAA gene serves multiple purposes. It confirms the diagnosis, especially in cases with atypical presentations, and provides information about inheritance patterns. Mutation analysis is also crucial for genetic counseling, enabling families to understand recurrence risks and consider reproductive options [32–35]. Furthermore, knowledge of the molecular defect enables the tailoring of emerging therapies, including gene therapy, to specific genotypes [21, 22].

In conclusion, the diverse spectrum of mutations in the GAA gene underpins the phenotypic variability observed in Pompe disease. Moreover, detailed molecular analysis is crucial for accurate diagnosis, prognosis, and informed therapeutic decision-making.

## 4 GENETIC COUNSELING

Genetic counseling is an integral component in the management of Pompe disease, particularly given its autosomal recessive inheritance and the significant variability in clinical presentation [36]. This process involves assessing familial risk, providing education about the genetic basis of the disease, and discussing reproductive options for affected families [37, 38].

Pompe disease occurs when an individual inherits pathogenic mutations in the GAA gene from both parents. Genetic counseling begins with a detailed family history to identify consanguinity, which significantly increases the risk of autosomal recessive conditions. In cases like the Moroccan family reported by Adadi *et al*. (2018), consanguinity was a critical factor in identifying high recurrence risk within the family [5].

Carrier testing for at-risk family members is a key aspect of genetic counseling. It allows the identification of heterozygous carriers, who typically do not exhibit symptoms but have a 50% chance of passing the mutated gene to their offspring.

Genetic counseling provides information about reproductive options for families at risk [36]. Prenatal genetic testing through chorionic villus sampling (CVS) or amniocentesis can determine whether a fetus has inherited two pathogenic GAA mutations [39, 40]. Preimplantation genetic diagnosis (PGD) offers an alternative for families undergoing *in vitro* fertilization, allowing the selection of embryos free from disease-causing mutations [17, 22].

Additionally, Ethnic and regional variations in GAA mutations necessitate population-specific counseling strategies. For individuals diagnosed with Pompe disease, counseling focuses on educating patients and families about the natural history of the disease, available treatments, and potential outcomes. This includes discussing therapeutic options like ERT and emerging gene therapies, as well as potential complications and their management [20, 41]. Counseling asymptomatic individuals, such as carriers, involves addressing emotional and psychological concerns, including implications for family planning.

The diagnosis of Pompe disease, particularly in infants and children, often carries a heavy emotional burden. Genetic counseling addresses these challenges by providing psychological support and facilitating informed decision-making. Ethical issues, including the decision to undergo prenatal testing or terminate affected pregnancies, are handled with sensitivity and respect for the family's cultural and personal values [16, 19].

In the Moroccan family studied by Adadi *et al*. (2018), genetic counseling played a pivotal role in guiding future reproductive decisions [5]. The identification of a homozygous GAA mutation not only confirmed the diagnosis but also enabled carrier testing for extended family members, reducing the likelihood of recurrence in subsequent generations. Thus, in conclusion, genetic counseling is crucial for empowering families with Pompe disease to make informed decisions, facilitate early diagnosis, and support emotional and psychosocial well-being.

## 5 THERAPIES

### 5.1 Enzyme Replacement Therapy (ERT)

ERT is the cornerstone of treatment for Pompe disease [1, 42]. This approach involves the intravenous administration of recombinant human alpha-glucosidase (rhGAA) to restore deficient enzyme activity and reduce glycogen accumulation in lysosomes [41, 43]. Two formulations, alglucosidase alfa (Myozyme^®^ and Lumizyme^®^), are currently approved for clinical use.

ERT delivers functional GAA enzymes to lysosomes *via* the mannose-6-phosphate receptor pathway. Once internalized, the enzyme breaks down accumulated glycogen, mitigating cellular damage and improving tissue function [13, 17].

ERT has significantly improved the survival and quality of life for patients with IOPD. Regular administration of rhGAA reduces cardiomyopathy, improves muscle strength, and delays the need for ventilatory support. However, its effectiveness in reversing skeletal muscle damage is limited due to poor enzyme uptake in this tissue [19].

In LOPD, ERT helps stabilize or slow the progression of muscle weakness and respiratory dysfunction. While the response is variable, patients often experience improved mobility and pulmonary function. The earlier the therapy is initiated, the better the outcomes [16, 21].

Nonetheless, ERT has several limitations, which are as follows:

1. **Immunogenicity:** Many patients, particularly those with cross-reactive immunologic material-negative (CRIM-negative) genotypes, develop antibodies against **rhGAA**. These antibodies may neutralize the enzyme's activity, reducing its efficacy [22].
2. **Limited skeletal muscle uptake:** Due to poor receptor availability and enzyme delivery efficiency, skeletal muscle damage is often incompletely addressed.
3. **High cost and accessibility:** ERT is expensive, with limited availability in resource-constrained settings, posing significant barriers to widespread adoption [5].

Efforts to enhance ERT include combining it with immune tolerance induction protocols to mitigate antibody formation. The development of next-generation enzymes with enhanced skeletal muscle uptake is also underway [19, 22].

A Moroccan infant diagnosed with IOPD *via* enzymatic and genetic testing began ERT with alglucosidase alfa. The therapy reduced cardiomyopathy and improved respiratory function, though skeletal muscle weakness persisted. This case underscores the benefits and limitations of ERT in severe phenotypes [5].

Additionally, advancements in ERT are focusing on modifying enzyme formulations to enhance delivery to affected tissues and on combining ERT with gene therapy for more comprehensive treatment. These approaches aim to overcome current limitations and improve long-term outcomes for patients with Pompe disease [13].

### 5.2 Gene Therapy

Gene therapy is an emerging treatment strategy for Pompe disease, aimed at addressing the limitations of ERT by introducing a functional GAA gene into patient cells [12]. This approach aims to restore the sustained, endogenous production of the enzyme GAA, potentially reducing glycogen accumulation and thereby mitigating disease progression.

Gene therapy utilizes viral vectors, predominantly adeno-associated viruses (AAVs), to deliver a functioning GAA gene to target cells. Once delivered, the transgene is expressed, enabling the production of functional GAA enzyme, which is then distributed systemically or taken up by lysosomes to break down glycogen [13, 19].

In preclinical studies, gene therapy has demonstrated significant efficacy in animal models of Pompe disease. Restoration of GAA activity has been associated with reduced glycogen storage in both muscle and cardiac tissues, as well as improved motor function. Current clinical trials are exploring the safety and efficacy of AAV-mediated GAA delivery in humans. Early-phase trials have shown promise, with sustained enzyme activity reported in treated patients [22].

Gene therapy offers several advantages compared to ERT:

1. **Sustained enzyme production:** Unlike ERT, which requires lifelong infusions, gene therapy has the potential for long-lasting therapeutic effects from a single administration.
2. **Reduced immunogenicity:** By enabling endogenous enzyme production, gene therapy minimizes the formation of neutralizing antibodies that can impair treatment efficacy.
3. **Improved skeletal muscle uptake:** Gene therapy enhances enzyme delivery to skeletal muscles, a key limitation of ERT [17].

Despite its promise, several challenges remain:

1. **Vector immunogenicity:** Immune responses to the viral vector can limit its effectiveness and may necessitate immune suppression during treatment.
2. **Targeting efficiency:** Achieving sufficient delivery to all affected tissues, including skeletal and respiratory muscles, remains a technical hurdle.
3. **Durability of expression:** Although gene therapy is designed for long-term correction, durability may vary depending on the vector and dosing strategy.
4. **Cost and accessibility:** Like ERT, gene therapy is anticipated to be highly expensive, raising concerns about equitable access [19, 22].

Nevertheless, combining gene therapy with ERT or immune-modulating strategies could further optimize treatment outcomes. For example, initial ERT could reduce glycogen accumulation, enhancing the efficacy of subsequent gene therapy.

Ongoing research is focused on improving vector design, enhancing transduction efficiency, and reducing immunogenicity. Efforts to develop non-viral gene delivery systems are also underway, which could overcome some of the current challenges [16].

In the context of severe infantile-onset Pompe disease, gene therapy has the potential to transform the therapeutic landscape. For families like the one reported by Adadi *et al*. (2018), gene therapy offers hope for a more effective and durable treatment, addressing the shortcomings of ERT and providing a pathway to better long-term outcomes [5]. Gene therapy represents a transformative approach to managing Pompe disease. Although still in its experimental stages, it holds promise for sustained therapeutic benefits and an improved quality of life for affected individuals.

## CONCLUSION

Pompe disease, a rare autosomal recessive lysosomal storage disorder caused by mutations in the *GAA* gene, presents a spectrum of clinical manifestations ranging from severe infantile-onset disease to milder late-onset forms. Advances in molecular diagnostics, including enzymatic assays and genetic testing, have significantly improved our ability to diagnose and characterize the disease, facilitating earlier and more accurate identification of affected individuals.

ERT has been transformative, particularly for infantile-onset Pompe disease, by extending survival and improving cardiac and respiratory function. However, its limitations, including suboptimal skeletal muscle penetration, immunogenicity, and the need for lifelong infusions, underscore the necessity for complementary or alternative therapies. Gene therapy, currently in experimental stages, holds significant promise as a durable treatment option, offering the potential for sustained enzyme activity and more comprehensive disease management.

Moreover, genetic counseling plays a vital role in addressing the hereditary nature of Pompe disease. It helps families understand recurrence risks, explore reproductive options, and navigate the emotional and psychosocial challenges associated with the condition. Region-specific genetic studies further support tailored approaches to diagnosis and care.

Future efforts must focus on improving the accessibility of therapies, refining gene therapy techniques, and exploring combination strategies that integrate ERT and gene therapy for synergistic effects. Research into the underlying mechanisms of disease variability and progression will also be crucial for developing precision medicine approaches. As exemplified by the Moroccan family studied by Adadi *et al*. (2018), Pompe disease remains a diagnostic and therapeutic challenge, particularly in resource-constrained settings. Continued advancements in research, coupled with efforts to reduce global disparities in access to care, will be essential to improving outcomes and quality of life for all patients affected by this complex disorder.

## AUTHORS’ CONTRIBUTIONS

N.A. conceived and designed the study and was responsible for data collection. N.A. and M.Y.E.B. contributed to the analysis and interpretation of the results. Both authors participated in drafting the manuscript. N.A. and M.Y.E.B. critically revised the content and approved the final version of the manuscript. All authors reviewed the results and approved the final version of the manuscript.

## Figures and Tables

**Table 1:** Studies supporting diagnostic approaches for pompe disease.

Aspect	Description	References
Enzymatic assays	Measures acid alpha-glucosidase (GAA) activity in blood, fibroblasts, or muscle tissue. Low activity confirms diagnosis.	[13, 21]
Genetic testing	Identifies mutations in the GAA gene to confirm enzymatic results and understand genotype-phenotype correlations.	[5, 22]
Biochemical markers	Elevated serum creatine kinase (CK), ALT, and AST levels indicate muscle damage and help monitor disease progression.	[17]
Electrodiagnostic studies	Electromyography (EMG) reveals myopathic changes but is not specific to Pompe disease.	[16]
Imaging modalities	MRI identifies muscle involvement, echocardiography detects hypertrophic cardiomyopathy, and pulmonary function tests assess diaphragmatic weakness.	[19, 20]
Case Study insight	A Moroccan family showed severe IOPD phenotype with reduced GAA activity and a homozygous mutation in the GAA gene, confirming the diagnosis.	[5]

## LIST OF ABBREVIATIONS

AAVs: Adeno-associated Viruses
ERT: Enzyme Replacement Therapy
GAA: Acid Alpha-Glucosidase
IOPD: Infantile-Onset Pompe Disease
LOPD: Late-Onset Pompe Disease
PGD: Preimplantation Genetic Diagnosis
rhGAA: Recombinant human Alpha-Glucosidase Acid
